# Distribution shift detection for the postmarket surveillance of medical AI algorithms: a retrospective simulation study

**DOI:** 10.1038/s41746-024-01085-w

**Published:** 2024-05-09

**Authors:** Lisa M. Koch, Christian F. Baumgartner, Philipp Berens

**Affiliations:** 1 Hertie Institute for AI in Brain Health, University of Tübingen, Tübingen, Germany; 2 Cluster of Excellence Machine Learning: New Perspectives for Science, University of Tübingen, Tübingen, Germany; 3 Faculty of Health Sciences and Medicine, University of Lucerne, Lucerne, Switzerland; 4 Tübingen AI Center, Tübingen, Germany

**Keywords:** Health care, Eye diseases

## Abstract

Distribution shifts remain a problem for the safe application of regulated medical AI systems, and may impact their real-world performance if undetected. Postmarket shifts can occur for example if algorithms developed on data from various acquisition settings and a heterogeneous population are predominantly applied in hospitals with lower quality data acquisition or other centre-specific acquisition factors, or where some ethnicities are over-represented. Therefore, distribution shift detection could be important for monitoring AI-based medical products during postmarket surveillance. We implemented and evaluated three deep-learning based shift detection techniques (classifier-based, deep kernel, and multiple univariate kolmogorov-smirnov tests) on simulated shifts in a dataset of 130’486 retinal images. We trained a deep learning classifier for diabetic retinopathy grading. We then simulated population shifts by changing the prevalence of patients’ sex, ethnicity, and co-morbidities, and example acquisition shifts by changes in image quality. We observed classification subgroup performance disparities w.r.t. image quality, patient sex, ethnicity and co-morbidity presence. The sensitivity at detecting referable diabetic retinopathy ranged from 0.50 to 0.79 for different ethnicities. This motivates the need for detecting shifts after deployment. Classifier-based tests performed best overall, with perfect detection rates for quality and co-morbidity subgroup shifts at a sample size of 1000. It was the only method to detect shifts in patient sex, but required large sample sizes ($> 30^{\prime} 000$). All methods identified easier-to-detect out-of-distribution shifts with small (≤300) sample sizes. We conclude that effective tools exist for detecting clinically relevant distribution shifts. In particular classifier-based tests can be easily implemented components in the post-market surveillance strategy of medical device manufacturers.

## Introduction

Machine learning (ML) tools for automated medical image interpretation have been approaching expert-level performance in controlled settings in recent years^1^. However, major hurdles still obstruct the wide adoption of machine learning in clinical practice. When ML is applied in clinical care, its outputs are often used to inform patient management decisions. Therefore, as a flipside to their vast potential, ML algorithms can also cause harm to the patient. This can happen if ML model failures provide incorrect information to treating physicians in the decision-making process. In a screening setting for example, if ML algorithms perform poorly in detecting early stages of a disease, patients may be treated inappropriately, leading to increased burden on public health systems overall.

ML systems are considered medical devices if they are intended and used for patient care, e.g. as defined in^2^ or the FD&C Act, Sec. 201(h). Strict regulations (e.g. by the FDA in the US or the recently applied Medical Device Regulation (MDR) in the European Union) facilitate their safety and effectiveness. Under these regulations, artificial intelligence (AI) algorithms for the automated analysis of retinal fundus images were among the first to be approved and to be commercially available^3^. The main use case of currently available products is screening for diabetic retinopathy, an eye disease occuring as a consequence of vascular damage caused by diabetes. The disease is highly prevalent among diabetics and a frequent source of blindness, which can be prevented through early screening, as this allows adjustments of the lifestyle, better control of blood glucose or targeted therapies. Therefore, high quality automated screening devices are of great interest.

**Fig. 1:**
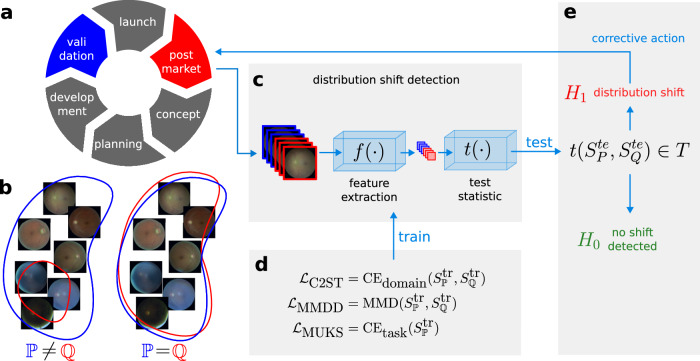
Overview of the distribution shift detection framework in the context of postmarket surveillance of a medical image-based ML device. **a** The medical device lifecycle including validation (blue) and postmarket (red) phases, (**b**) illustration of potential distribution shifts between the source distribution ${\mathbb{P}}$ (blue) and the target ${\mathbb{Q}}$ (red), where all images from ${\mathbb{Q}}$ are within the support of the source distribution and thus cannot be detected as outliers. **c** Subgroup and other distribution shifts can be detected using neural network-based hypothesis tests consisting of a deep feature extractor and a test statistic. **d** The feature extractors and their optimisation depend on the shift detection approach. **e** Finally, a hypothesis test is carried out on held-out test samples. If a shift is detected, its causes can be investigated and corrective action can be taken in the context of post-market activities of a medical device manufacturer.

Medical device regulations govern the whole life cycle of a product (Fig. 1a), with the validation phase playing a key role in the evaluation of AI-based clinical tools. The validation phase includes AI model and software verification and testing, full product testing and usability evaluation, and is typically followed by a clinical validation, where the performance is assessed on data that are intended to be representative of the real data distribution encountered by the deployed system. After clinical validation, the system may be declared safe (e.g. by the manufacturer) and approved for use in the intended setting. “Safe” in this context means that the benefits of using the ML system are considered to outweigh the risks associated with prediction errors. This benefit-risk analysis crucially hinges on realistic ML performance estimates: if the performance in the deployment setting falls short of the claimed performance estimated in the validation setting, the cumulative harm associated with prediction errors in all patients may exceed the level deemed acceptable, and specifically, the level reported during validation.

To demonstrate that a medical device stays within the validated performance specifications during real-life use, device manufacturers are required to implement post-market surveillance (PMS) measures that continuously monitor the deployed system during the postmarket phase (highlighted in red in Fig. 1a). Regulatory bodies and manufacturers are increasingly seeking and adopting stronger PMS components. In the US, this is for example reflected in the strategy outlined in^4^, the recently proposed guiding principles for good ML practice^5^ and the predetermined change control plans for ML-enabled medical devices^6^. In Europe, this process is for example reflected in stricter PMS requirements in the MDR. Effective post-market surveillance is of particular importance for opaque deep learning systems, where failure mechanisms are difficult to inspect and changes over time in ML systems’ input data and outputs may therefore go unnoticed. Nevertheless, efficient techniques for detecting post-market changes in the distribution of data used by a certified medical product have received considerably little attention in the research community.

In real settings, such changes in performance can be caused by a myriad of factors that induce data distribution shifts with respect to the validation setting. Examples include changes in technology (for example affecting low-level characteristics of acquired images), changes in patient demographics or changes in behaviour (for example increased screening for a disease in specific demographics due to new guidelines)^7^. Many of these changes can be subtle and difficult to detect by humans, but may lead to unexpected changes in performance^8^.

One type of dataset shift that is particularly difficult to detect is subgroup shift. Subgroup shifts can occur when the prevalence of individual subgroups is different in real-world data encountered by the deployed algorithm compared to the clinical validation data^9^. For example, an algorithm for screening for diabetic retinopathy may be evaluated on a heterogeneous dataset spanning multiple sites, various imaging protocols and devices, and including a diverse patient population. While validation on heterogeneous data is in principle highly desirable, there likely is (hidden) stratification in the data, and performance in subgroups may vary distinctly^10,11^. It is possible that the ML system is then predominantly applied to a subgroup of the original population: this can happen if the ML system is deployed to screen patients in hospitals with lower-quality imaging procedures or other subsets in acquisition technology, or hospitals with a catchment area predominantly inhabited by members of certain ethnicities. Such factors lead to a distribution shift which can impact the real-world performance, or will at least render original performance claims invalid. Both scenarios pose serious problems for safely deploying machine learning systems. While a shift could be detected by measuring and monitoring the distribution of patient attributes and acquisition settings, crucial covariates characterising relevant subgroup attributes are often unmeasured or unidentified^10–12^. The individual images from the (shifted) deployment data distribution resemble data from a subgroup of the original data. No individual data point of the deployment data is therefore atypical or “out-of-distribution” (OOD) compared to the original distribution. Conventional outlier detection methods^13–18^ aim to identify images that are unlikely under the original data distribution, and are therefore unfortunately unsuitable for detecting such hidden subgroup shifts^9^.

In this paper, we address the problem of detecting clinically relevant distribution shifts in retinal imaging data acquired in a diabetic retinopathy screening and monitoring setting from multiple hospitals with highly varying demographics, and argue that our solution can be employed as part of the post-market surveillance strategy of a medical device manufacturer also in other domains. Related work so far either relied on subgroup labels for training robust models^19–22^, or aimed at identifying relevant stratifications as a preliminary step which could facilitate downstream intervention^10–12^. In contrast, our proposed approach is agnostic of metadata and operates directly and solely on the input images by detecting whether two groups of images are systematically different. We argue that subgroup shifts can be detected as distribution shifts at deployment stage. To detect shifts, we propose to use the framework of statistical hypothesis testing with a null hypothesis that the dataset that the classifier was trained and validated on and the dataset it is applied to are drawn from the same distribution. Recently developed deep learning-based hypothesis tests have reached meaningful statistical power on high-dimensional data^23–27^, but this problem has so far not been explored in a medical imaging setting. This study builds on our own prior work^9^, where we presented a proof of concept in a toy setting. Here, we focus on the following key goals:

1. We hypothesise that the performance of a deep learning model for the task of diabetic retinopathy detection varies between population subgroups in a multi-hospital real-world dataset of fundus images, motivating the need for detecting distribution shifts during post-market surveillance.
2. We seek an overview of hypothesis testing approaches that are applicable to distribution shift detection in high-dimensional imaging data, including the detection of subgroup shifts.

## Results

### Subgroup performance disparities for diabetic retinopathy grading

We trained a deep learning model for diabetic retinopathy (DR) detection on a real-world, ethnically diverse and multi-hospital dataset. We obtained a 5-class DR grading accuracy on the test set of 0.87. The accuracy varied strongly for the different DR grades, with poor performance for the severely underrepresented classes. We then also evaluated the classifier’s performance at detecting referable DR, which is clinically useful in a screening setting and routinely done to assess DR grading models^28^. Referable DR is defined as a DR grade of 2 or above, thus we grouped the healthy and mild cases (grades 0, 1) and the moderate or more severe classes (grades 2, 3, 4). We found considerable performance differences across subgroups in all reported metrics (right column in Table 1). For example, the referable DR detection performance for different ethnicities ranged between 0.50 and 0.79 sensitivity, and classification performance in all metrics was reduced for images with lower quality. An undetected distribution shift in the deployed ML system towards a higher prevalence of these patients could lead to increased patient harm. Furthermore, we found a considerable accuracy drop for the group of patients with co-morbidities. Interestingly, for this group the sensitivity at detecting referable DR increased, indicating that in the presence of co-morbidities, differentiating between diseases may be challenging.

**Table 1:** Summary of relevant patient and image attributes in the dataset, along with their prevalence in the training, validation and test folds

		Number of images									
Attribute	Group	Training		Val.		Test		5-acc	2-acc	2-sens	2-spec
		*N*	%	*N*	%	*N*	%				
All		78126		26210		26150		0.87	0.94	0.66	0.98
Sex	Female	46684	60	15795	60	15641	60	0.88	0.94	0.66	0.98
	Male	31418	40	10413	40	10501	40	0.85	0.93	0.66	0.98
	Other	24	0	2	0	8	0	0.88	1.00	-	1.00
	African Descent	6057	8	2006	8	1979	8	0.83	0.91	0.63	0.98
	Asian	4362	6	1572	6	1273	5	0.90	0.95	0.60	0.98
	White	8648	11	2929	11	2977	11	0.90	0.95	0.54	0.99
Ethnicity	Indian	3675	5	1356	5	1212	5	0.81	0.90	0.79	0.96
	Latin American	54011	69	17908	68	18251	70	0.87	0.94	0.66	0.98
	Multi-racial	769	1	160	1	197	1	0.85	0.91	0.66	0.97
	Native American	604	1	279	1	261	1	0.82	0.88	0.50	0.96
Image quality	Excellent	14321	18	4892	19	4884	19	0.89	0.96	0.67	0.99
	Good	32184	41	10712	41	10719	41	0.88	0.94	0.66	0.98
	Adequate	31621	40	10606	40	10547	40	0.84	0.92	0.66	0.97
Co-morbidities	Not present	70370	90	23512	90	23595	90	0.87	0.94	0.58	0.98
	Present	7756	10	2698	10	2555	10	0.84	0.90	0.82	0.96
DR grade	0	61330	79	20702	79	20561	79	0.99	-	-	-
	1	5589	7	1802	7	1882	7	0.12	-	-	-
	2	9806	13	3289	13	3290	13	0.63	-	-	-
	3	631	1	204	1	189	1	0.04	-	-	-
	4	770	1	213	1	228	1	0.19	-	-	-

The rightmost columns show DR grading performance in subgroups of the test set: the accuracy of the finegrained classification problem with 5 DR grades (5-acc) and the evaluation of its performance in the binary task of detecting referable DR in terms of accuracy (2-acc), sensitivity (2-sens), and specificity (2-spec).

### Classifier-based tests reliably detect distribution shifts in retinal images

**Fig. 2:**
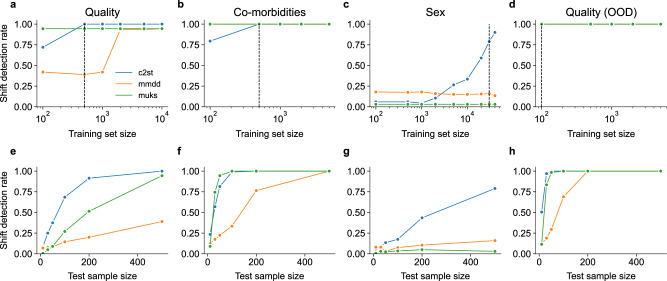
Shift detection rate for subgroup shifts. Panels show subgroup shifts with respect to (**a**) image quality, (**b**) the presence of co-morbidities, (**c**) patient sex. **d** Shows the shift detection rate for an out-of-distribution shift to images of insufficient quality. The top row (**a–d**) shows the shift detection rate for a fixed test sample size (*m* = 500) when varying the size of the training set. In the bottom row (**e–h**), the training dataset size was fixed (see dashed line in the respective plot above), and the test sample size varied between *m* = 10 and *m* = 500.

We simulated distribution shifts with respect to patient sex, ethnicity, the presence of co-morbidities, and image quality (see Sec. Simulating distribution shifts). For each shift type, we analysed the shift detection rates of the detection methods C2ST, MMDD and MUKS. For a given training set size *n*_train_ drawn from the respective source and target distributions, we trained the shift detection models C2ST, MMDD (see Sec.. Technical details on methods for distribution shift detection), and then evaluated the shift detection rate on separate test sets of size *m* = 500 (Fig. 2a–d). The MUKS test only requires access to the available DR detection model, and no additional training data for the shift detection model itself.

The classifier-based test C2ST performed best at detecting a wide range of distribution shifts. C2ST required only 500 training examples for detecting quality and co-morbidity subgroup shifts with perfect detection rate (dashed vertical lines in Fig. 2a, b). The MMDD and MUKS test performed on par with C2ST for detecting co-morbidity shifts, but in particular MMDD fell short for shifts in image quality. Furthermore, C2ST was the only method to successfully detect subgroup shifts in patient sex, but required ${n}_{{{{\rm{train}}}}}=30^{\prime} 000$ training images to reach a detection rate of 0.79 (Fig. 2c). In contrast to image quality and co-morbidities, patient sex is is not visibly discernible in retinal images, which could explain the inferior shift detection for this more subtle shift type.

To analyse the tradeoff of training and test sample size, we fixed the training dataset size (bottom row of Fig. 2, see dashed line in respective plot above) and analysed the sensitivity of the test power to the test sample size for the different shift detection techniques and shift types. As expected, the shift detection rate decreased with fewer test samples, but C2ST remained relatively robust for a quality subgroup shifts. For co-morbidity shifts, the detection rate remained perfect both C2ST and MUKS while decreasing the test sample size to *m* = 100. For a subgroup shift in patient sex (Fig. 2g), all methods decreased with decreasing test sample size.

Finally, we also studied an out-of-distribution shift, where the target distribution contained lower quality images than were present in the source data. For such a shift, all methods reached perfect test power with only 100 training examples, and even the test sample size could be reduced drastically to ca. *m* = 50 for C2ST and MUKS and *m* = 200 for MMDD. So to summarise, to detect this out-of-distribution shift, only a total of 100 + 50 = 150 images were necessary from each distribution, indicating this was a much easier task.

**Fig. 3:**
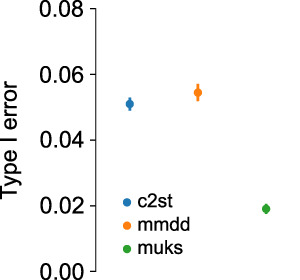
Type I error for all shift detection methods, averaged over all shift types. The error bar depicts the standard deviation.

For all tests, the type I error (i.e. the false positive detection rate) remained approximately within the chosen significance level (Fig. 3).

### Detection rate of ethnicity shifts is increased for ethnic minorities

**Fig. 4:**
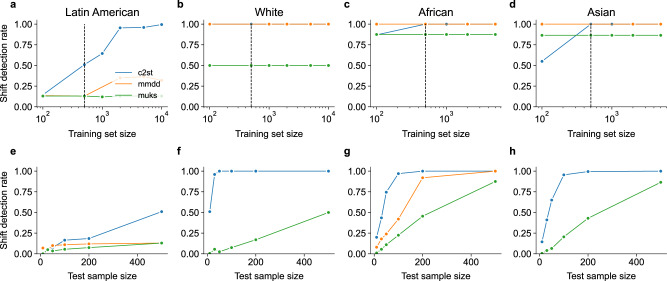
Shift detection rate for subgroup shifts towards different ethnicites. The top row (**a–d**) shows the shift detection rate for a fixed test sample size (*m* = 500) when varying the size of the training set. In the bottom row (**e–h**), the training dataset size was fixed (see dashed line in the respective plot above), and the test sample size varied between *m* = 10 and *m* = 500.

We next examined subgroup shifts in patient ethnicity. Here, we examined the subgroups of Latin American, White, African and Asian patients, which were present in the source distribution with decreasing frequency (see Table 1). We simulated each shift by including only patients who self-report as belonging to these individual ethnicities in the target distribution. All shift detection methods performed better for the subgroups with lower prevalence in the source distribution (Fig. 4a–e). C2ST and MMDD reached perfect shift detection performance for White, African and Asian subgroup shifts even with only *n*_train_ = 100 (White) or *n*_train_ = 500 (African and Asian) training examples. We argue that subgroup shifts towards underrepresented subgroups resemble out-of-distribution shifts, which could explain the strong shift detection performance Table 2.

**Table 2:** Influence of input image resolution on C2ST and MMDD test power for subgroup shifts in retinal images

Shift		Test sample size					
		10	30	50	100	200	500
	C2ST-512	0.36	0.85	0.97	1.00	1.00	1.00
Co-morbidities	C2ST-96	0.17	0.45	0.64	0.94	1.00	1.00
	MMDD-96	0.11	0.31	0.26	0.40	0.79	1.00
	C2ST-512	0.39	0.63	0.72	0.84	0.88	0.93
Ethnicity	C2ST-96	0.38	0.60	0.68	0.78	0.82	0.89
	MMDD-96	0.11	0.34	0.34	0.35	0.70	1.00
	C2ST-512	0.14	0.29	0.47	0.81	0.96	1.00
Quality	C2ST-96	0.07	0.14	0.23	0.45	0.66	0.98
	MMDD-96	0.08	0.11	0.12	0.15	0.15	0.42
	C2ST-512	0.06	0.07	0.04	0.08	0.06	0.04
Sex	C2ST-96	0.06	0.04	0.05	0.07	0.07	0.07
	MMDD-96	0.08	0.08	0.09	0.12	0.14	0.18

For this analysis, we set the training set size to *n*_train_ = 1000.

On the other hand, Latin American patients constituted a majority of the source distribution, therefore a subgroup shift to Latin American patients only was more subtle and more difficult to detect (Fig. 4a). Still, the classifier-based test C2ST approached close to perfect detection when using *n*_train_ = 2000 training images. However, MMDD and MUKS were not able to detect this subtle distribution shift.

### Influence of image resolution on test power

It is well-known that automated DR grading requires high resolution fundus images^29^. This motivated the use of 512 × 512 sized images wherever possible. For the MMDD method, we reduced the image size to 96 × 96 because calculating the kernel-based test statistic for this method was too memory-intensive otherwise. To study whether the large performance gap between MMDD and C2ST could be explained by the reduced image size for MMDD, we evaluated C2ST at lower resolution (C2ST-96) as well for a number of shift types and a fixed training set size of *n*_train_ = 1000. Overall, C2ST-96 consistently outperformed MMDD-96, indicating that image resolution was not a key reason for MMDD’s inferior performance. The notable exception was a shift in patient sex, where test power of the classifier-based test was highly sensitive to image size.

### Influence of architecture on classifier-based tests

To further probe the behaviour of classifier-based tests, we studied their robustness to less powerful domain classifier architectures using quality subgroup shifts as an example setting. We replaced the original ResNet-50 backbone in the domain classifier network with a ResNet-18 and also with a shallow model consisting of four convolutional layers similar to the architecture used in MMDD. Test power was reduced in both cases (Table 3). As expected, degradation was more notable with a shallow domain classifier, while the ResNet-18 only led to minor reductions in test power.

**Table 3:** Influence of the domain classifier architecture on test power for a quality shift in retinal fundus images

	Test sample size					
	10	30	50	100	200	500
C2ST-ResNet-50	0.14	0.29	0.47	0.81	0.96	1.00
C2ST-ResNet-18	0.09	0.20	0.41	0.74	0.96	0.99
C2ST-Shallow	0.04	0.08	0.08	0.06	0.13	0.18

The training set size was set to *n*_train_ = 1000.

### Sensitivity to subgroup shift strength

**Fig. 5:**
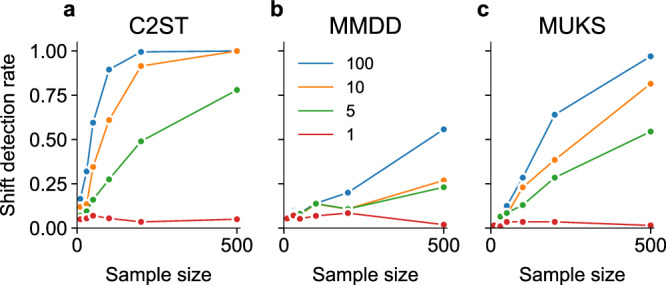
Influence of varying quality subgroup shift strength on test power of the different shift detection techniques. Panels show (**a**) C2ST, (**b**) MMDD, and (**c**) MUKS tests.

Finally, we examined the sensitivity of our subgroup shift detection tests to only subtle changes between distributions ${\mathbb{P}}$ and ${\mathbb{Q}}$. To control the subgroup shift strength, we artificially adjusted the prevalence of low quality images. We sampled with replacement from the whole dataset, and gradually over-represented these images with factors *w* = {1, 5, 10, 100} in ${\mathbb{Q}}$ compared to ${\mathbb{P}}$, and again set the training set size to *n*_train_ = 1000. As expected, stronger shifts consistently led to higher detection rate (Fig. 5). Classifier-based tests were more robust to small decreased shift strengths, while performance drops were more severe for MMDD and MUKS. No oversampling (*w* = 1, blue curves) implied ${\mathbb{P}}={\mathbb{Q}}$, and there test power expectedly remained in line with the chosen significance level.

## Discussion

We performed a retrospective simulation study on the usefulness of distribution shift detection methods for a variety of shifts in a real-world, ethnically diverse retinal image dataset. We showed that subgroup shifts such as a shift in image quality or patient ethnicity can indeed affect the overall performance of a ML system for DR grading. In particular, the sensitivity at detecting referable DR varied severely between 0.50 and 0.79 for different ethnic subgroups. Such performance disparities are particularly concerning when medical AI is used in global operations where devices may be deployed outside the narrow confines of clinically validated settings. Overall, the classification model we trained for demonstration purposes yielded a relatively a low sensitivity on our dataset, compared to previously reported values on public datasets such as the Kaggle Diabetic Retinopathy challenge^30^. In our work, we relied on the chosen dataset because of the availability of rich metadata, and trained the DR grading model using state-of-the-art recommendations^29^. The model in its current state would not be suitable for deployment and should be recalibrated with a more clinically suitable sensitivity-specificity tradeoff. Developing an application-ready DR grading model was, however, not the goal of this work. Rather, it served the purpose of motivating and studying our proposed shift detection methods.

We then adopted three state-of-the-art approaches towards detecting subgroup shifts in medical images. All approaches used the framework of statistical hypothesis testing for shift detection and relied on neural network based representations of the images for meaningful test statistics. Our experiments showed that in particular classifier-based tests (C2ST) consistently and considerably outperformed the other approaches.

In our earlier work^9^, we had omitted C2ST from our experiments as^25^ had found them to be inferior to deep kernel tests for detecting distribution shifts on less complex data distributions. The results we reported here contradict these previous findings. We suspect that C2ST yielded superior power because the C2ST test relies on training a “vanilla” classification task, and as a community, we have converged to a good understanding of suitable engineering choices for solving such tasks. In contrast, training a kernel test requires maximising the maximum mean discrepancy between two minibatches, and more exploration may be needed to solve this task robustly.

Multiple univariate Kolmogorov-Smirnov tests^24^ yielded lower test power in most shift types. Partly, the inferior performance could be explained by the conservative Bonferroni correction for multiple comparisons, which we used here in line with^24^. Since MUKS tests rely on a task classifier as a feature extractor, MUKS performance is by design tied to systematic changes in task predictions. The MUKS method therefore seemed to work reasonably well in scenarios such as co-morbidity shifts, where it is plausible that task predictions were affected. While not ideal in terms of general test power, MUKS tests have the advantage that they do not require training data from the deployment distribution.

The shift detection methods described in this article can in principle be readily implemented in the postmarket surveillance system of a manufacturer, but some practical questions remain before real-world application. Depending on the availability of real-world data, MUKS may be more applicable than C2ST and MMDD, as MUKS does not require training any model on real-world deployment data. However, as C2ST and MMDD tests are completely task-agnostic and do not require labelled data for training, the availability of real-world data should not pose a hurdle in many settings.

In this article, we relied on labelled attributes that were available to us to simulate distribution shift settings, as this was actionable for the purpose of demonstrating the power of our method. The simulated scenarios do not comprehensively capture the diversity of shifts that may be encountered in real applications. For example, we only studied image quality as one possible acquisition factor while there are a myriad of changes in technology or personnel training that may affect image acquisition. However, our method should be agnostic to the precise source and nature of shifts, with the caveat that stronger or more distinct shifts are more easily detectable. We emphasise that the proposed tools will be applied in a setting where we do not know or measure the attributes underlying a change in the deployment setting. Rather, our approach requires only access to unlabelled data from two distributions (i.e. validation and deployment data).

An important limitation of this work is the fact that a distribution shift will not necessarily lead to poor performance, and this cautious approach to postmarket monitoring goes beyond common practice in pre-ML medical devices. Therefore, detecting shifts is not sufficient in itself for monitoring the performance of the deployed system. However, our approach can reveal unexpected changes and trigger an investigation of their root cause and potential consequences as outlined in Sec. Perspective: Application within a regulatory framework. In this work, we have optimised the shift detection rate at a certain threshold to prevent “false positive” detections, i.e. alerts when no actual distribution shift happened. The current threshold was at 5% false positive rate (Type I error) in line with the practice in the technical literature on neural-network based shift detection. Future work may show that our method may be fairly sensitive in real-world scenarios, and thresholds may be adjusted when implementing our approach for a PMS system.

Overall, we have shown that effective tools exist for detecting clinically relevant distribution shifts, and we see the proposed techniques as useful and easily implemented components in the post-market surveillance strategy of medical device manufacturers. As a final step before implementing these measures in a live PMS system, we recommend to evaluate our approach on historical data of encountered distribution shifts of a deployed AI system in collaboration with a medical AI device manufacturer. If the postmarket monitoring were carried out by an external auditor rather than the manufacturer themselves, implementing the shift detection techniques proposed here may be more difficult as they require technical domain knowledge specific to the product. However, at least under the Medical Device Regulation in the EU, implementing PMS measures is the responsibility of the manufacturer, thus making our approach feasible in practice.

## Methods

### Dataset description and data preparation

**Fig. 6:**
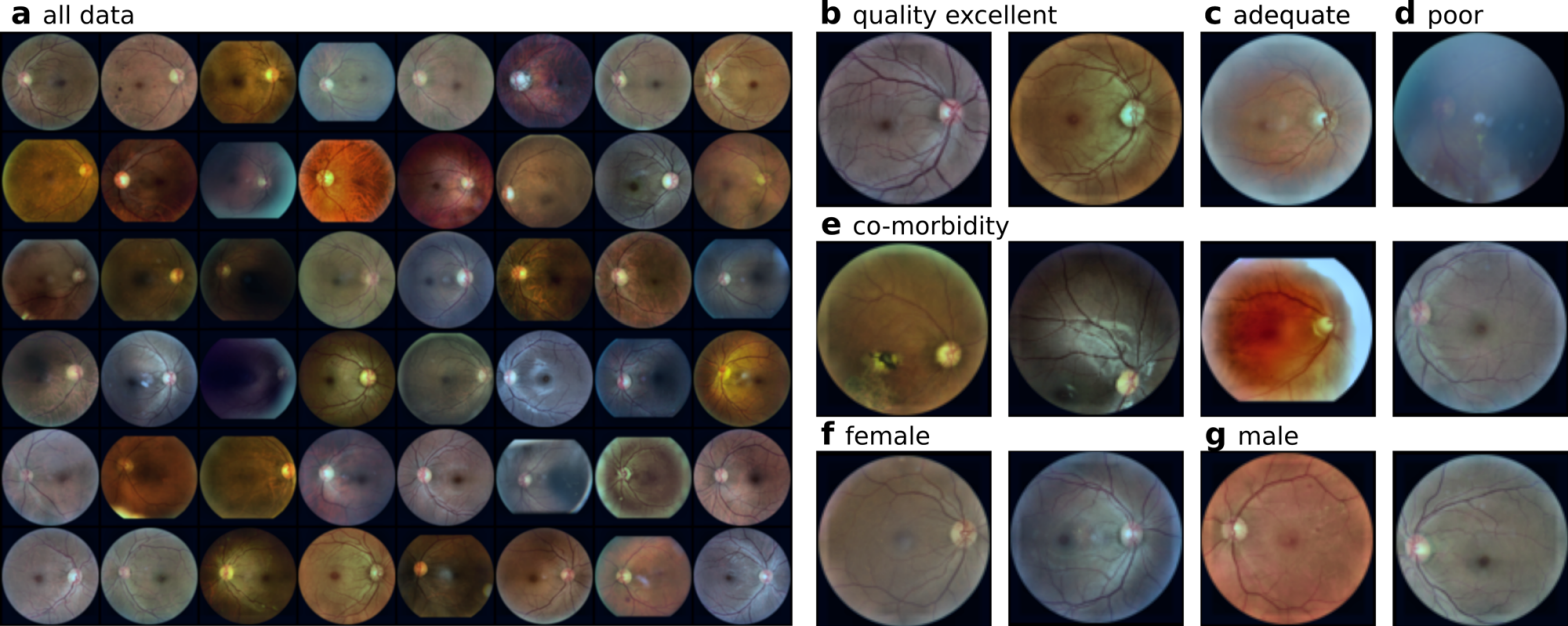
Example retinal fundus images from the Eyepacs dataset. Panel (**a**) shows a random selection. Magnified on the right are example (**b**) images of excellent, (**c**) adequate and (**d**) poor quality, (**e**) images with co-morbidities, and example images of (**f**) female and (**g**) male patients. For better visibility, the example images were contrast enhanced using contrast limited adaptive histogram equalisation (CLAHE).

We used an anonymised real-world, ethnically diverse multi-hospital retinal fundus dataset from EyePACS, Inc collected in California, USA. The dataset consisted of 186’266 macula-centred images from 44’135 patients. We seeked approval by the ethics committee of the medical faculty of the University of Tübingen (project number 121/2024BO2) and consent was waived as anonymised data are exempt from mandatory counselling. We only included images which where manually assessed to be of at least adequate image quality for interpretation (91% of the images), and which contained DR grades (available for 99% of the images) and self-reported information on patient sex (97%) and ethnicity (77%). Combining these restrictions resulted in 130’486 macula-centred images from 31’805 patients (see examples in Fig. 6a). Furthermore, beyond expert DR grades, the presence of co-morbidities was reported including eye diseases such as cataract, glaucoma and others. The data were split into training (78’126), validation (26’210) and test (26’150) folds. Table 1 provides a detailed overview of the included population with relevant patient and image attributes. The dataset was very imbalanced w.r.t. DR grades (bottom rows in Table 1) and contained 79% healthy images (grade 0) and only approximately 2% severe cases (grades 3, 4). Different image or patient attributes can visibly impact fundus image appearance, such as image quality or the presence of co-morbidities (Fig. 6b–e). In contrast, patient sex (**f**-**g**) cannot be visually inferred from the image by human experts. We used this dataset to study potentially harmful subgroup shift scenarios where the real-world distribution may consist of subgroups of the population, e.g. a changing prevalence of patients with existing co-morbidities or specific ethnicity, or data with lower image quality only. While we focused mainly on the detection of subgroup shifts, the studied hypothesis test also lend themselves for out-of-distribution shifts, i.e. when individual images from the target data are fundamentally different from the source data. To study this setting, we used an additional “out-of-distribution” set of 7’767 (4751 training / 1512 validation / 1504 test) very low quality images which were deemed insufficient for interpretation. For these images, DR grading performance could not be assessed, but a shift from the original data distribution to this low quality dataset would be harmful if undetected.

All images were pre-processed by cropping a square bounding box fitting the circular fundus and resampling the images to a resolution of 512 × 512 pixels.

### Deep learning model for diabetic retinopathy grading

We trained a deep learning model that classified fundus images in 5 DR grades, ranging from 0 (healthy) to 4 (proliferative DR). The model was implemented in PyTorch v1.10. We used a ResNet-50 neural network architecture^31^ and a cross-entropy loss. The loss was optimised using stochastic gradient descent with Nesterov momentum, an initial learning rate (LR) of 0.001 and LR decay of 0.9. Data augmentation included random cropping, horizontal and vertical flipping, colour distortion and rotation. The model was trained for 25 epochs, and the final model was chosen based on the validation loss. The classifier was trained on the training fold of the source distribution, and then evaluated in individual subgroups.

### Deep learning pipeline for distribution shift detection

We introduce a general monitoring framework which can serve as a tool during the postmarket surveillance of deployed medical AI algorithms (Fig. 1). Our goal was to detect distribution shifts between the validation and postmarket (i.e. deployment) phases of a medical AI device (Fig. 1a, b). We assume a clinically validated image-based ML algorithm such as the one trained for diabetic retinopathy detection in this study, and investigate the scenario where the manufacturer (or an auditor) has access to data from the validation and the real-world postmarket phase. We refer to these as the source and target distribution ${\mathbb{P}}$ and ${\mathbb{Q}}$, respectively. Following^32,33^, we formulate the problem of distribution shift detection as a hypothesis test with null hypothesis ${H}_{0}:{\mathbb{P}}={\mathbb{Q}}$ and alternative ${H}_{1}:{\mathbb{P}}\,\ne\, {\mathbb{Q}}$.

A useful hypothesis test for distribution shift detection should have a high test power or detection rate, i.e. a high probability that *H*_0_ is correctly rejected if the source and target distributions are indeed different. Classical statistical tests typically rely on strong assumptions on the parameters of the data distribution or are not suitable for high-dimensional data^25^. For high-dimensional data such as images, an effective hypothesis test could therefore either use low-dimensional representations of the images^24^, or use more flexible test statistics (e.g. ref.^33^). Both strategies could be approached using conventional methods, but neural-network based hypothesis tests^24–26^ have outperformed these and have recently led to high test power on toy image datasets. Essentially, neural-network based hypothesis tests^24–26^ calculate a test statistic from learned feature representations of the high-dimensional image data in which the two domains ${\mathbb{P}}$ and ${\mathbb{Q}}$ are well separated. Based on our own prior work^9^ and a performance comparison on toy data in^24,25^, we have identified three hypothesis testing frameworks as suitable candidates for detecting subgroup distribution shifts in medical images.

In a *classifier-based test* (C2ST), a deep learning model is explicitly trained to discriminate between examples from ${\mathbb{P}}$ and ${\mathbb{Q}}$ and its output is used as a test statistic^26,27^. Alternatively, *Deep Kernel Tests* (MMDD) use trainable deep kernel functions and the distance metric maximum mean discrepancy between probability distributions as a test statistic^25,33^. Finally, *multiple univariate Kolmogorov-Smirnov tests on task predictions* (MUKS) uses the output of the monitored ML algorithm as a low-dimensional representation of the data together with univariate distribution shift tests for detecting subgroup shifts during deployment. We provide mathematical details in Sec. Technical details on methods for distribution shift detection. For all hypothesis tests, we set the significance level to *α* = 0.05 as in^24–26^. This corresponds to a 5% false positive rate (Type I error) of detecting a shift when none occurred in practice.

The procedure for applying such shift detection tests consists of the following steps, assuming we have access to a sample from the source distribution and a sample from the target distribution:

1. Split both the source and the target data into train and test folds.
2. Fit the parameters of the neural-network based feature extractors on the training fold ${S}_{{\mathbb{P}}}^{{{{\rm{tr}}}}},{S}_{{\mathbb{Q}}}^{{{{\rm{tr}}}}}$ of both the source and target distributions (Fig. 1c, d).
3. Perform a two-sided hypothesis test on the deep features of the test fold ${S}_{{\mathbb{P}}}^{{{{\rm{te}}}}},{S}_{{\mathbb{Q}}}^{{{{\rm{te}}}}}$ from the source and target data (Fig. 1e).

If a shift is detected, this would indicate that the deployment setting of the ML algorithm may violate its intended use. An alert could be issued and corrective action could be taken by the manufacturer to investigate the reason and implications of the observed shift (Fig. 1e). The integration of our approach into a product’s postmarket surveillance system within a regulatory framework is described in more depth in Sec. Perspective: Application within a regulatory framework.

### Perspective: application within a regulatory framework

While this paper mostly focuses on the technical aspects of the proposed distribution shift techniques, here we briefly outline how these methods could be used within the context of a regulatory framework. As an application scenario, we use the example of a DR detection tool developed and deployed as Software as a Medical Device (SaMD) under the Medical Device Regulation. The manufacturer of such a device is required to “plan, establish, document, implement, maintain and update a post-market surveillance system” as an integral component of its quality management system (Art. 83(1) MDR). The PMS system should monitor the performance and safety of a device during its lifetime such that it can identify and implement necessary preventive and corrective action (Art. 83(4) MDR).

As part of its PMS system, the manufacturer will collect real-life data that were used as an input by the DR detection tool. This requires technical access to the data itself as well as careful consideration of data protection. Using the methods described in this paper, the manufacturer will then periodically check for distribution shifts w.r.t the clinical validation data. Here, the manufacturer is responsible for technically implementing these monitoring techniques rather than an external auditor. This makes the practical application of our approach straightforward, as the manufacturer of an ML-based DR detection tool will have the technical infrastructure and experience to implement the neural-network based shift detectors on retinal images.

If a distribution shift is detected, this will issue an alert and start the processes outlined in the manufacturer’s postmarket surveillance plan (Art. 84 MDR). These processes are typically implemented in a Corrective and Preventive Action (CAPA) system. The manufacturer will investigate the potential root causes of the shift, for example: could there have been a change in demographics or a change in technology? Importantly, not every distribution shift requires corrective action. The shift’s potential consequences will be investigated to update the benefit-risk analysis (Art. 83(3) MDR), asking questions such as: Is the system’s intended use violated? Could the distribution shift lead to a performance drop? If the manufacturer determines that corrective action is ncecessary, they implement the appropriate measures and inform the competent authorities and notified bodies (Art. 83(4) MDR). All detected distribution shift events and the results of the follow-up investigations, including any preventive or corrective measures, will be documented and the reports made available to the notified bodies (Art. 85/86 MDR).

### Data requirements for the studied methods

The studied shift detection methods use training data for learning feature extractors to compute test statistics, and then perform the hypothesis tests on test data (see Table 4). C2ST and MMDD require training images from both source and target distributions, but they require no disease labels. In contrast, MUKS does not require access to the target distribution for training at all. Instead, it uses images from the source distribution with ground truth labels for the monitored ML algorithm’s task. None of the methods require task labels from the target distribution. While the real-world data availability may depend on the specific application and deployment route of a medical device, we argue that all three methods can be applicable in postmarket surveillance procedures, where no labels can be expected, but access to unlabelled target data should be available.

**Table 4:** Data requirements for the studied distribution shift detection methods

Method	Train		Test	
	Images	Labels	Images	Labels
C2ST	${\mathbb{P}},{\mathbb{Q}}$	–	${\mathbb{P}},{\mathbb{Q}}$	–
MMDD	${\mathbb{P}},{\mathbb{Q}}$	–	${\mathbb{P}},{\mathbb{Q}}$	–
MUKS	${\mathbb{P}}$	${\mathbb{P}}$	${\mathbb{P}},{\mathbb{Q}}$	–

### Simulating distribution shifts

We studied distribution shifts with respect to patient sex, ethnicity, the presence of co-morbidities, and image quality. To model shifts in the prevalence of co-morbidities, we included only the subgroup of images with co-morbidities in the target distribution. For image quality, we simulated a subgroup shift by considering only “Adequate” images in the target distribution ${\mathbb{Q}}$, compared to all images of either adequate, good, or excellent quality in ${\mathbb{P}}$. We also simulated an out-of-distribution shift in quality, where the target distribution consisted of images of insufficient quality. This class of images was not present at all in the source distribution. For both quality and co-morbidity shift, we observed considerable performance drops in these subgroups (see Table 1), making such distribution shifts potentially harmful if undetected. Furthermore, we argue that attributes such as image quality and presence of co-morbidities may not be routinely assessed in clinical validation settings and even less in deployment settings, and such shifts could thus go unnoticed unless detected based on the image distributions directly. We also modelled shifts in patient sex by including only images from female patients in the target distribution, and ethnicity shifts by individually including only a single ethnicity.

### Evaluation criteria

Our main evaluation criterion is the shift detection rate, which corresponds to the test power in statistical hypothesis testing: it denotes the rate at which a distribution shift is correctly detected, i.e. the probability that *H*_0_ is correctly rejected if *H*_1_ is true. The shift detection rate can be calculated by repeatedly drawing samples from the source and target dataset, applying the shift detection method, and calculating the proportion of repetitions where a shift was detected, i.e. *H*_0_ was rejected.

While the shift detection rate should be as high as possible, false positives should be low. We therefore also report the false positive rate. The false positive rate denotes the proportion of repetitions where a shift was wrongly detected, i.e. when the source and target distributions match. The false positive rate corresponds to the type I error in statistical hypothesis testing. It can be calculated by applying repeated tests to samples drawn from the source distribution only.

### Technical details on methods for distribution shift detection

In this section, we explain the technical aspects of the studied deep hypothesis tests in more detail. We refer to the source and target distributions as ${\mathbb{P}}$ and ${\mathbb{Q}}$ over ${{{\mathcal{X}}}}$ and ${{{\mathcal{Y}}}}$, respectively. ${{{\mathcal{X}}}}\subseteq {{\mathbb{R}}}^{n},{{{\mathcal{Y}}}}\subseteq {{\mathbb{R}}}^{n}$ are subsets of the space of *n*-dimensional images. We formally define a subgroup shift as the scenario where ${{{\mathcal{Y}}}}\subseteq {{{\mathcal{X}}}}$ and ${\mathbb{P}}\,\ne\, {\mathbb{Q}}$. Following^32,33^, we formulate the problem of subgroup shift detection as a hypothesis test with null hypothesis ${H}_{0}:{\mathbb{P}}={\mathbb{Q}}$ and alternative ${H}_{1}:{\mathbb{P}}\,\ne\, {\mathbb{Q}}$. In statistical hypothesis testing, the hypotheses make a statement about a population parameter, and the test statistic *t*(*X*, *Y*) is the corresponding estimate from the samples $X={\{{x}_{i}\}}_{i = 0}^{m}\mathop{ \sim }\limits^{iid}{\mathbb{P}}$ and $Y={\{{y}_{i}\}}_{i = 0}^{m}\mathop{ \sim }\limits^{iid}{\mathbb{Q}}$. *H*_0_ is rejected for some rejection region *T* of *t*.

Essentially, neural-network based hypothesis tests^24–26^ rely on feature representations of the high-dimensional image data in which the two domains ${\mathbb{P}}$ and ${\mathbb{Q}}$ are well separated. The procedure for applying such a test consists of two steps: We first fit the parameters of the neural-network based feature extractors on a training fold ${S}_{{\mathbb{P}}}^{{{{\rm{tr}}}}},{S}_{{\mathbb{Q}}}^{{{{\rm{tr}}}}}$ of both the source and target distributions, and then perform a hypothesis test on the deep features of the test fold ${S}_{{\mathbb{P}}}^{{{{\rm{te}}}}},{S}_{{\mathbb{Q}}}^{{{{\rm{te}}}}}$ from the source and target data.

*Classifier-based hypothesis tests (C2ST)* make use of a domain classifier that aims to discriminate between examples from ${\mathbb{P}}$ and ${\mathbb{Q}}$^26,27^. As domain classification becomes impossible for ${\mathbb{P}}={\mathbb{Q}}$, this motivates using a measure based on the classification performance as a test statistic *t*(*X*, *Y*). The null hypothesis could then be rejected in favour of ${H}_{1}:{\mathbb{P}}\,\ne\, {\mathbb{Q}}$ for significantly above chance performance^26^. proposed a test statistic based on the classifier’s logit output ${f}_{{{{\rm{C2ST}}}}}:{{{\mathcal{X}}}}\to {\mathbb{R}}$:

1 $$t(X,Y)=\frac{1}{m}\mathop{\sum}\limits_{i}{f}_{{{{\rm{C2ST}}}}}({x}_{i})-\frac{1}{m}\mathop{\sum}\limits_{j}{f}_{{{{\rm{C2ST}}}}}({y}_{j})$$

Thus, the domain classifier can be viewed as a feature extractor whose parameters are fit on training images from the source and target distribution ${S}_{{\mathbb{P}}}^{{{{\rm{tr}}}}},{S}_{{\mathbb{Q}}}^{{{{\rm{tr}}}}}$, using for example a cross-entropy loss to distinguish examples from ${\mathbb{P}}$ and ${\mathbb{Q}}$. Fitting the parameters of the C2ST feature extractor therefore requires only knowledge of the domain membership, but no labels with regard to a specific task. The test statistic from Eq. (1) can be calculated on two held out samples from the source and training data ${S}_{{\mathbb{P}}}^{{{{\rm{te}}}}},{S}_{{\mathbb{Q}}}^{{{{\rm{te}}}}}$ and a permutation test can be carried out to determine the rejection threshold.

For the application studied in this paper, we trained a domain classifier *f*_C2ST_ using a binary ResNet-50 and the same training configuration as the task classifier described in Sec. Deep learning model for diabetic retinopathy grading.

*Deep kernel tests (MMDD)*^25^ are a recent generalisation of kernel two-sample tests^33^, which use a distance metric between probability distributions as a test statistic to examine whether ${H}_{0}:{\mathbb{P}}={\mathbb{Q}}$. Intuitively, a suitable distance metric should be low if the null hypothesis is true (i.e. the distributions ${\mathbb{P}},{\mathbb{Q}}$ are the same), and higher for ${\mathbb{P}}\,\ne\, {\mathbb{Q}}$, thus allowing us to reliably reject *H*_0_. One such a distance metric is the Maximum Mean Discrepancy (MMD) on a Reproducing Kernel Hilbert Space (RKHS)^33^:

As in^33^, let ${{{{\mathcal{H}}}}}_{k}$ be a RKHS with kernel $k:{{{\mathcal{X}}}}\,\times\, {{{\mathcal{X}}}}\to {\mathbb{R}}$. The MMD is defined as

2 $${{{\rm{MMD}}}}[{{{{\mathcal{H}}}}}_{k},{\mathbb{P}},{\mathbb{Q}}]=\mathop{\sup }\limits_{f\in {{{{\mathcal{H}}}}}_{k},{\left\Vert f\right\Vert }_{{{{{\mathcal{H}}}}}_{k}}\le 1}\left({{\mathbb{E}}}_{x \sim {\mathbb{P}}}[f(x)]-{{\mathbb{E}}}_{y \sim {\mathbb{Q}}}[f(y)]\right),$$

and an unbiased estimator for the MMD is

3 $$\widehat{{{{\rm{MMD}}}}}(X,Y;k)=\frac{1}{m(m-1)}\mathop{\sum}\limits_{i\ne j}{H}_{ij},$$

4 $${H}_{ij}=k({x}_{i},{x}_{j})+k({y}_{i},{y}_{j})-k({x}_{i},{y}_{j})-k({y}_{i},{x}_{j})$$

For characteristic kernels *k*, the MMD is a metric, which implies that ${{{\rm{MMD}}}}[{{{{\mathcal{H}}}}}_{k},{\mathbb{P}},{\mathbb{Q}}]=0$ iff ${\mathbb{P}}={\mathbb{Q}}$. The metric property makes $t(X,Y)=\widehat{{{{\rm{MMD}}}}}(X,Y)$ an appropriate test statistic for testing whether ${\mathbb{P}}={\mathbb{Q}}$.

The choice of the kernel *k* affects the test power in finite sample sizes and developing suitable kernels is an active area of research (e.g. ref. ^25,34^). We follow^25^ and parameterise the kernel *k* with a neural network. Specifically^25^, use a neural network ${f}_{{{{\rm{MMDD}}}}}:{{{\mathcal{X}}}}\to {{\mathbb{R}}}^{128}$ as a feature extractor and define the final kernel as a combination between two Gaussians kernels operating on the original image space and the feature space, respectively:

5 $$k(x,y)=\left((1-\delta ){g}_{a}(f(x),f(y))+\delta \right){g}_{b}(x,y),$$

where *g*_*a*_, *g*_*b*_ are Gaussians with length scales *σ*_*a*_, *σ*_*b*_. The kernel parameters, including neural network parameters *θ*, are optimised on training images from ${\mathbb{P}}$ and ${\mathbb{Q}}$ by maximising the Maximum Mean Discrepancy:

6 $${{{{\mathcal{L}}}}}_{{{{\rm{MMDD}}}}}=-\widehat{{{{\rm{MMD}}}}}({S}_{{\mathbb{P}}}^{{{{\rm{tr}}}}},{S}_{{\mathbb{Q}}}^{{{{\rm{tr}}}}};k)$$

We diverge slightly from^25^, where the objective function also incorporated knowledge on the asymptotic distribution of $\widehat{{{{\rm{MMD}}}}}$ under *H*_1_. We did not find this beneficial, as reported in our prior work^9^. For a trained kernel, the test statistic $t(X,Y)=\widehat{{{{\rm{MMD}}}}}(X,Y)$ can be calculated on samples *X*, *Y* from the test fold of the source and target data ${S}_{{\mathbb{P}}}^{{{{\rm{te}}}}},{S}_{{\mathbb{Q}}}^{{{{\rm{te}}}}}$, and again a permutation test can be used to determine whether ${H}_{0}:{\mathbb{P}}={\mathbb{Q}}$ can be rejected.

For applying MMDD to retinal images, we reduced the image size from 512 × 512 to 96 × 96 as the original resolution was prohibitive for calculating the MMDD test statistic, which contained pairwise terms (Eq. (3)), for large sample sizes. Furthermore, due to the poor shift detection performance of MMDD on retinal images in preliminary experiments, we replaced the shallow convolutional network in the original feature extractor *f*_MMDD_^25^ with a ResNet-50 model with a 128-dimensional output.

Finally, *multiple univariate Kolmogorov-Smirnov (MUKS) tests*^24^ are hypothesis tests for domain shift detection that operate on low-dimensional representations of the original image space ${{{\mathcal{X}}}}$. They use a black-box task classifier ${f}_{{{{\rm{MUKS}}}}}:{{{\mathcal{X}}}}\to {{\mathbb{R}}}^{C}$ as a dimensionality reduction technique, where $s=f(x)\in {{\mathbb{R}}}^{C}$ is a softmax prediction for *x* with *C* classes. In our case *f*_MUKS_ can be the monitored ML algorithm itself. For simplicity, we train *f*_MUKS_ using images from ${\mathbb{P}}$ and associated labels.

Multiple univariate Kolmogorov-Smirnov (MUKS) test are then applied to the softmax predictions of samples *X* and *Y* from the test fold ${S}_{{\mathbb{P}}}^{{{{\rm{te}}}}},{S}_{{\mathbb{Q}}}^{{{{\rm{te}}}}}$. The two-sample KS test statistic can be calculated for each class individually as

7 $${t}_{c}(X,Y)=\mathop{\sup }\limits_{{f}_{c}(x)}| {F}_{X,c}({f}_{c}(x))-{F}_{Y,c}({f}_{c}(x))| ,$$

where *F*_*X*,*c*_, *F*_*Y*,*c*_ are the empirical distribution functions over the softmax outputs of samples *X*, *Y*. Standard KS tests can then be carried out, yielding *Cp*-values. As *C* tests are performed, the *p*-values must be corrected for multiple comparisons. As in ^24^, we perform Bonferroni correction, i.e. *H*_0_ is rejected if *p* ≤ *α*/*C* for any of the *C* comparisons.

### Reporting summary

Further information on research design is available in the Nature Research Reporting Summary linked to this article.

### Supplementary information

Reporting Summary

## Data Availability

The data used in this study were provided by EyePACS, Inc, a third party provider. Data are available from EyePacs Inc. (contact@eyepacs.org) upon request for a fee.

## Code availability

The code for training and evaluating the models used in this work is available at https://github.com/lmkoch/postmarket-shift-detection.
